# Altered Th17/Treg Ratio in Peripheral Blood of Systemic Lupus Erythematosus but Not Primary Antiphospholipid Syndrome

**DOI:** 10.3389/fimmu.2019.00391

**Published:** 2019-03-06

**Authors:** Lorena Álvarez-Rodríguez, Víctor Martínez-Taboada, Jaime Calvo-Alén, Iñaki Beares, Ignacio Villa, Marcos López-Hoyos

**Affiliations:** ^1^Transplantation and Autoimmunity Laboratory, Rheumatology Department, University Hospital Marqués de Valdecilla-IDIVAL, Santander, Spain; ^2^Faculty of Medicine, Rheumatology Department, University Hospital Marqués de Valdecilla-IDIVAL, Cantabria University, Santander, Spain; ^3^Rheumatology Department, University Hospital Araba, Vitoria-Gasteiz, Spain; ^4^Rheumatology Department, Hospital Sierrallana, Torrelavega, Spain; ^5^Immunology Department, University Hospital Marqués de Valdecilla-IDIVAL, Cantabria University, Santander, Spain

**Keywords:** pAPS, SLE, Th1, Th17, Treg, inflammation

## Abstract

**Introduction:** The role of the immune response in the pathogenesis of antiphospholipid syndrome (APS) remains elusive. It is possible that differences in the frequencies of Th17 cells and/or defects in the immunoregulatory mechanisms are involved in the pathogenesis of APS. Our aim was to determine the peripheral blood Th cells phenotype and the circulating cytokine profile in patients with primary APS (pAPS) and compare it with systemic lupus erythemathosus (SLE) as disease control group.

**Methods:** The frequencies of circulating regulatory T cells (Tregs) were determined in PBMCs from 36 patients with pAPS by flow cytometry. As control groups we included 21 age- and gender-matched healthy controls (HC) and 11 patients with SLE. The suppressive capacity of Tregs was evaluated *in vitro* by coculture assay. On the other hand, intracellular cytokine production was assessed in Th1, Th2, and Th17 cells and circulating IL-6, IL-10, and IL-35 were measured by Cytometric Bead Array and ELISA. The quantification of Th master gene expression levels was performed by real time quantitative PCR.

**Results:** pAPS patients and SLE patients did not show differences in the percentage or number of Tregs compared to HC. The suppressive capacity of Tregs was also similar in the three study group. Instead, we found higher FoxP3·mRNA expression levels in pAPS patients and HC than SLE patients. Regarding the Th17 response, patients with pAPS and HC showed a significantly lower frequency of circulating Th17 cells than SLE. However, no differences were observed in the Th1 response between patients and controls. Thus, increased Th17/Th1 and Th17/Treg ratios were found in SLE patients but not in pAPS patients. pAPS and SLE patients had higher serum IL-6 levels than HC but there was not difference between both disease groups. Besides, a significant increase in the immunosuppressive cytokine levels was observed only in pAPS as compared to HC.

**Conclusions:** Our data demonstrate an increased inflammatory profile of peripheral blood CD4^+^ T cells from SLE as compared with pAPS mostly due to an increased Th17 response. In conclusion, there seems not to be a direct pathogenic role for Th cells in pAPS but in SLE.

## Introduction

The antiphospholipid syndrome (APS) is a systemic autoimmune inflammatory disease characterized by the presence of serum antiphospholipid antibodies (aPL) and clinically by vascular thrombosis and/or obstetric events (1). Several lines of evidence indicate that aPL contribute to the inflammatory response that plays an important role in the pathogenesis of APS (2, 3). Thus, aPL have also emerged as triggers of innate immune inflammatory pathways in APS, through the activation of toll like receptors (TLR) (4). Besides, some authors have described that aPL might induce *in vitro* Th2 and Th17, but not Th1 or Treg differentiation (5). Although a clear association between aPL and clinical manifestations in APS has been established, the pathogenesis of this syndrome is poorly understood and it is generally considered multifactorial with an inflammatory background (6).

On the other hand, the clinical distinction between primary APS (pAPS) and APS secondary to other autoimmune disorders is sometimes difficult, especially with APS secondary to systemic lupus erythematosus (SLE) (7). APS and SLE share some clinical features, such as multiorgan manifestations, and the aPL profile and complement activation. At the same time, there are differences in the serum autoantibodies detected in both syndromes and we have recently demonstrated that the B cell phenotype differs between pAPS and SLE (8). From a pathogenic point of view, both entities are inflammatory disorders in which the activation of the innate immune response triggers an exacerbated acquired immune response (9). SLE and pAPS share an IFN-inducible gene expression signature (10). Besides, peripheral blood mononuclear B cells from pAPS patients show an additional monocyte-elicited inflammatory response demonstrated by increased expression of genes such as TLR8 and CD14 (11). In any case, both are inflammatory processes that drive autoantibodies production with some differences, and it is possible that the different expression of inflammatory genes may induce different T cells responses (7). However, there are not studies addressing differences in the circulating numbers or function of all the main T CD4+ subsets in pAPS, such as Th1, Th2, Th17, and Tregs, which play important roles in autoimmune diseases. Differences in the distribution of these cells may favor the hypotheses for differentiating SLE from pAPS.

Apart from the differences between pAPS and SLE, there is another important issue regarding the two main clinical variants of APS: the thrombotic and the obstetric APS (12). The differentiation between both variants may give and therapeutic potential. Since, no different autoantibodies have been found to distinguish vascular and obstetric events, differences in the circulating T cell phenotype might help. Although there are scarce works evaluating the peripheral blood T cell phenotype in pAPS (13, 14), to our knowledge there is no study comparing it between the vascular and obstetric variants.

In this study, we analyzed in the peripheral blood compartment the frequencies of Th1, Th2, Th17, and Tregs in patients with pAPS and compared them with SLE patients and healthy controls (HC). Besides, we addressed whether there was any difference for these cells between pAPS with thrombotic manifestations and obstetric disorders.

## Materials and Methods

### Patients

The present study included 36 patients with pAPS (17 patients with obstetric complications and 19 patients with thrombotic phenomena), 11 patients with SLE, and 21 age-matched healthy controls (HC) without a previous history of infectious, neoplastic or autoimmune disease. Patients with pAPS were diagnosed according to the Sydney classification criteria (1). aPL were considered positive when medium and high titers of serum aPL were confirmed in two separate determinations at least 12 weeks. The cut off value was set at our laboratory in 20 GPL and MPL for anti-cardiolipin antibodies and 20 U/ml for anti-β2GPI antibodies (Aeskulab, Wendelsheim, Germany). pAPS patients were considered as a whole and divided into vascular and obstetric subtypes. The obstetric complications were those of classification APS criteria (9 patients had one or more early pregnancy losses, 12 patients had late pregnancy loss, and three patients had a live birth with prematurity) and in two cases non-classification criteria were found (intrauterine growth restriction). Patients with SLE had to satisfy the ACR 1997 and SLICC 2012 classification criteria (15, 16). All SLE patients were in remission or presented low disease activity defined by a score ≤4 in the systemic lupus erythematosus disease activity index (SLEDAI). The main demographic, clinical, and laboratory characteristics of the study population are shown in Table 1. All the patients and controls gave signed informed consent in accordance with the Declaration of Helsinki, and the study was approved by the Regional Ethics Committee.

**Table 1:** Demographic and main clinical features of patients with primary antiphospholipid syndrome (pAPS), systemic lupus erythematosus (SLE), and healthy controls (HC).

	**HC**	**pAPS**	**Obstetric APS**	**Thrombotic APS**	**SLE**
Number of patients, *n*	21	36	17	19	11
Age, years (mean ± SD)	40.29 ± 11.6	36.36 ± 9.8	34.5 ± 4.2	38.0 ± 12.8	32.8 ± 13.1
Females, *n* (%)	15 (71.4)	30 (83.3)	17 (100)	13 (68.4)	11 (100)
Follow-up, months (mean ± SD)	–	–	66.3 ± 22.5	77.7 ± 65.0	121.1 ± 72.5
Clinical manifestations associated with APS, *n* (%)	0	36 (100)	17 (100)	19 (100)	0
Obstetrical events, *n* (%)	–	17 (47.2)	17 (100)	0 (0)	–
Arterial thrombosis, *n* (%)	–	12 (33.3)	0 (0)	12 (63.2)	–
Venous thrombosis, *n* (%)	–	7 (19.4)	0 (0)	7 (36.8)	–
Positive aPL Serology, *n* (%)	0	36 (100)	17 (100)	19 (100)	6 (54.5)
Positivity for one Ab, *n* (%)	–	19 (52.8)	11 (64.7)	8 (42.1)	4 (36.3)
Positivity for two Ab, *n* (%)	–	11 (30.5)	5 (29.4)	6 (31.6)	0 (0)
Positivity for three Ab, *n* (%)	–	6 (16.7)	1 (5.9)	5 (26.3)	2 (18.2)
**SEROLOGICAL PROFILE**					
aCL, *n*(%)	–	29 (78.4)	15 (88.2)	14 (73.7)	5 (45.5)
aβGPI, *n* (%)	–	17 (45.9)	7 (41.2)	10 (52.6)	2 (18.2)
Lupus Anticoagulant, *n* (%)	–	15 (40.5)	3 (17.6)	12 (63.2)	3 (27.3)
**TREATMENT**					
Antiaggregant *n* (%)	–	23 (63.9)	16 (94.1)	7 (36.8)	4 (36.4)
Anticoagulant *n* (%)	–	15 (41.7)	0 (0)	15 (78.9)	1 (9.1)
Corticosteroids *n* (%)	–	3 (8.3)	0 (0)	3 (15.8)	3 (27.3)
Antimalarials *n* (%)	–	4 (11.1)	1 (5.9)	3 (15.8)	9 (81.8)

*Patients with pAPS were divided into the two groups considered: obstetric and thrombotic*.

*APS, antiphospholipid syndrome; aPL, antiphospholipid; SLE, Systemic lupus erythematosus; SD, standard deviation; aCL, anticardiolipin; aB2GPI, anti beta 2 glycoprotein; Ab, antibody*.

### Detection of Intracellular Cytokines in Circulating Lymphocytes and Monocytes by Flow Cytometry Analysis

Intracellular cytokine staining is able to detect the production and accumulation of cytokines in the endoplasmic reticulum following stimulation. Cells collected in sodium heparin tubes were polyclonally stimulated for 4 h with phorbol 12- myristate 13-acetate (PMA) (Sigma Aldrich, St Louis, Missouri, USA) and ionomycin (Calbiochem, Gibbstown, New Jersey, USA) in polystyrene tubes (lymphocytes) in the presence of brefeldin A (Sigma Aldrich).

After culture, cells were stained with PerCP-conjugated anti-CD3 (Clone SK7) antibody (BD Biosciences) to identify T lymphocytes. Thereafter, the red blood cells were lysed with FACS lysing solution (BD Biosciences), and the mononuclear cells were permeabilized using FACS Permeabilizing Solution (BD Biosciences) and intracellularly stained with FITC- or PE-conjugated cytokine-specific monoclonal antibodies (BD Biosciences) for IL-2, IFNγ, and IL-4 for lymphocytes (Clones: IL-2: 5344.11; IFN-γ: 25723.11; IL-4: 3010.211). For characterization of Th17 cells, cells were stimulated under the same conditions, followed by surface staining with anti-CD4 APC (clone SK3), anti-CD161 PerCP-Cy 5.5 (clone DX12), and/or anti-CCR6 PE (clone 11A9) and intracellular staining with anti-IFNγ-PE (clone 25723.11) and anti-IL-17 FITC (clone eBio64Dec17). BD Biosciences provided all the antibodies except Alexa Fluor 488-conjugated anti-IL-17 monoclonal antibody that was provided by eBiosciences.

The quantification of circulating regulatory T cells (CD4^+^ CD25^hi^ CD127^−/low^ CD27^+^ CD62L^+^ CD45RO^+^ FoxP3^+^, and CD8^+^CD28^−^CD27^+^) was performed by flow cytometry with the following specific monoclonal antibodies: CD3 (clone SK7), CD4 (SK3), CD8 (SK1), CD25 (clone 2A3), CD27 (clone M-T271), CD28 (clone, L293), CD45RO (clone UCHL1), CD62L (clone SK11), CD127 (clone hIL-7R-M21) (BD Biosciences), and FoxP3 (Clone PCH101) (eBiosciences). FoxP3 expression was analyzed by flow cytometry after intracellular staining using the APC- anti-human FoxP3 staining set (eBiosciences) and following the protocol recommended by the manufacturer.

Levels of intracellular cytokine-producing cells together with surface expression were determined by FACS Canto II Flow Cytometer (BD Biosciences) and analyzed using FACS Diva software (BD Biosciences) to quantify the numbers of the different T CD4^+^ subsets. Percentages of Th subsets were referred to the CD4^+^ T cells.

### Detection of Soluble Cytokines in serum

#### Cytometric Bead Array

The serum was isolated from 4 ml of blood obtained in tubes without additives from each individual and stored at −80°C until analysis. The quantitative determination of inflammatory cytokines in serum was performed using the Cytometric Bead Array (CBA) Human Th1/Th2/Th17 Cytokine kit (BD Biosciences; San Diego, California, USA). This kit allows quantitatively measure interleukin (IL) 2, IL-4, IL-6, IL-10, Tumor Necrosis Factor (TNF) α, Interferon (IFN) γ, and IL-17A protein levels in a single sample. The fluorescence produced by CBA beads was measured on a FACS Canto II Flow Cytometer (BD Biosciences) and analyzed using FCAP array software (Soft Flow Inc; New Brighton, MN, USA). Detection limits were 2.6 pg/ml for IL-2, 4.9 pg/ml for IL-4, 2.4 pg/ml for IL-6, 4.5 pg/ml for IL-10, 3.8 pg/ml for TNF-α, 3.7 pg/ml for IFN-γ, and 18.9 pg/ml for IL-17A.

#### ELISA

Serum levels of IL-35 were determined using the commercial human ELISA kit (USCN Life Science Inc, Wuha, China) in accordance with the manufacturer's instructions. The sensitivity of the ELISA kit for IL-35 was 6.0 pg/ml.

### Proliferation Assays

To evaluate the *in vitro* suppressive capacity of Treg toward effector T (Teff) cells, FACS-sorted Treg and Teff cells of HC (*n* = 9), pAPS (*n* = 12), and SLE patients (*n* = 5) were setup in a coculture assay. PBMCS from sodium-heparinized peripheral blood were obtained by Ficoll Histopaque 1077 (Sigma Aldrich) gradient centrifugation. PBMCs were stained with specific monoclonal antibodies (BD Biosciences): anti-CD25-PE (Clone 2A3), anti-CD4-PerCP (Clone SK3), and anti-CD127-Alexa fluor 647 (Clone hIL-7R-M21) for isolation by FACS sorting of two populations: regulatory T cells and effector T cells, defined as CD4^+^ CD25^hi^ CD127^−/low^ and CD4^+^ CD25^−^ CD127^+^, respectively. Purity of FACS-sorted CD4^+^ CD25^hi^ CD127^−/low^ T cells was routinely >97%. Following sorting, effector T cells were labeled with 10 μM CFSE (Vybrant CFDA SE Cell Tracer Kit; Molecular Probes, Eugene, Oregon, USA) in PBS-1% FBS buffer for 10 min at 37°C. Cells were washed and resuspended in culture medium at 5 × 10^5^ cells/ml. CFSE-labeled effector T cells were cultured *in vitro* (96-well round bottom plates) alone and cocultured with unlabelled regulatory T cells (1:1). Cells were either unstimulated or stimulated with aCD3+aCD28 (Dynabeads human T-activator CD3/CD28 (Invitrogen Dynal, Oslo, Norway) at a bead to-cell ratio 1:1 and incubated at 37°C and 5% CO_2_. After 4 days of culture, cells were harvested, and stained for surface markers and CFSE signal of gated lymphocytes was analyzed by flow cytometry using FlowJo Software. Cells were acquired in a FACS Canto II Flow Cytometer (BD Biosciences). The suppressive capacity of Tregs toward effector T cells in culture [ratio (1:1)/ Effect] was expressed as the proliferation index.

### Real Time Quantitative PCR

Quantification of *T-bet, GATA-3, FoxP3*, and *ROR*γ*t* gene expression levels in PBMCs from patients and healthy subjects was performed by real time quantitative PCR. Total RNA was extracted using Trizol Reagent (Invitrogen, Carlsbad, CA, USA) and phenol/chloroform extractions method. The RNA concentration was quantified with a NanoDrop spectrophotometer (Thermo Scientific, Wilmington, DE, USA) and RNA integrity was tested by electrophoresis on 1.5% agarose gel. cDNAs were obtained by retrotranscription using iScript cDNA synthesis Kit (Bio-Rad Laboratories, CA, USA) according to the manufacturer's recommendations in a Master Cycler Pro S thermal cycler (Eppendorf, Hamburg; Germany).

The primer sequences are the following:

**Table:** 

T***-bet***	forward 5′-GATGCGCCAGGAAGTTTCAT-3′, reverse 5′-GCACAATCATCTGGGTCACATT-3′;
***GATA-3***	forward 5′-CCCTCATTAAGCCCAAGCGA -3′, reverse 5′-GTCTGACAGTTCGCACAGGA -3′;
***FoxP3***	forward 5′-TTCAAGTTCCACAACATGCG ACCC -3′, reverse 5′-GCACAAAGCACTTGTGCAGACTCA-3′;
***ROR*γ***t*	forward 5′-CAGTCATGAGAACACAAATTGA AGTG -3′, reverse 5′-CAGGTGATAACCCCGTAGTGGAT -3.
The housekeeping gene β-actin was used as an endogenous control,	
	forward 5′-ACCAACTGGGACGACATGGAGAAA -3′, reverse 5′-TAGCACAGCCTGGATAGCAACGTA-3′.

Real-time quantitative PCR was carried out using the Sso Fast Evagreen Supermix (Bio-Rad) according to the manufacturer's recommendations. PCR reactions were conducted in duplicate in a CFX96 detection system (Bio-Rad) and raw data were converted into the threshold cycle (Ct) values for each sample. Relative quantification was analyzed by the comparative Ct method, also referred to as 2^−ΔΔCt^ method, described by Schmittgen and Livak (17). The *p*- values were calculated based on a Student's *t*-test for each gene in patients and healthy subjects.

### Statistical Analysis

The normality was assessed using the Shapiro Wilk test. The data from the healthy controls and patient groups were first analyzed by Kruskall-Wallis test. The statistical comparisons of data between different pathologies and healthy controls were performed using the Mann-Whitney *U*-test. Correlations were assessed using Spearman's rank correlation coefficient. Differences were considered significant when *p* values were < 0.05. All the statistical analysis of data was carried out with the SPSS 15.0 software (Chicago, Illinois, USA).

## Results

### Circulating T Cells With Regulatory Phenotype in Patients With pAPS and SLE

**Figure 1:**
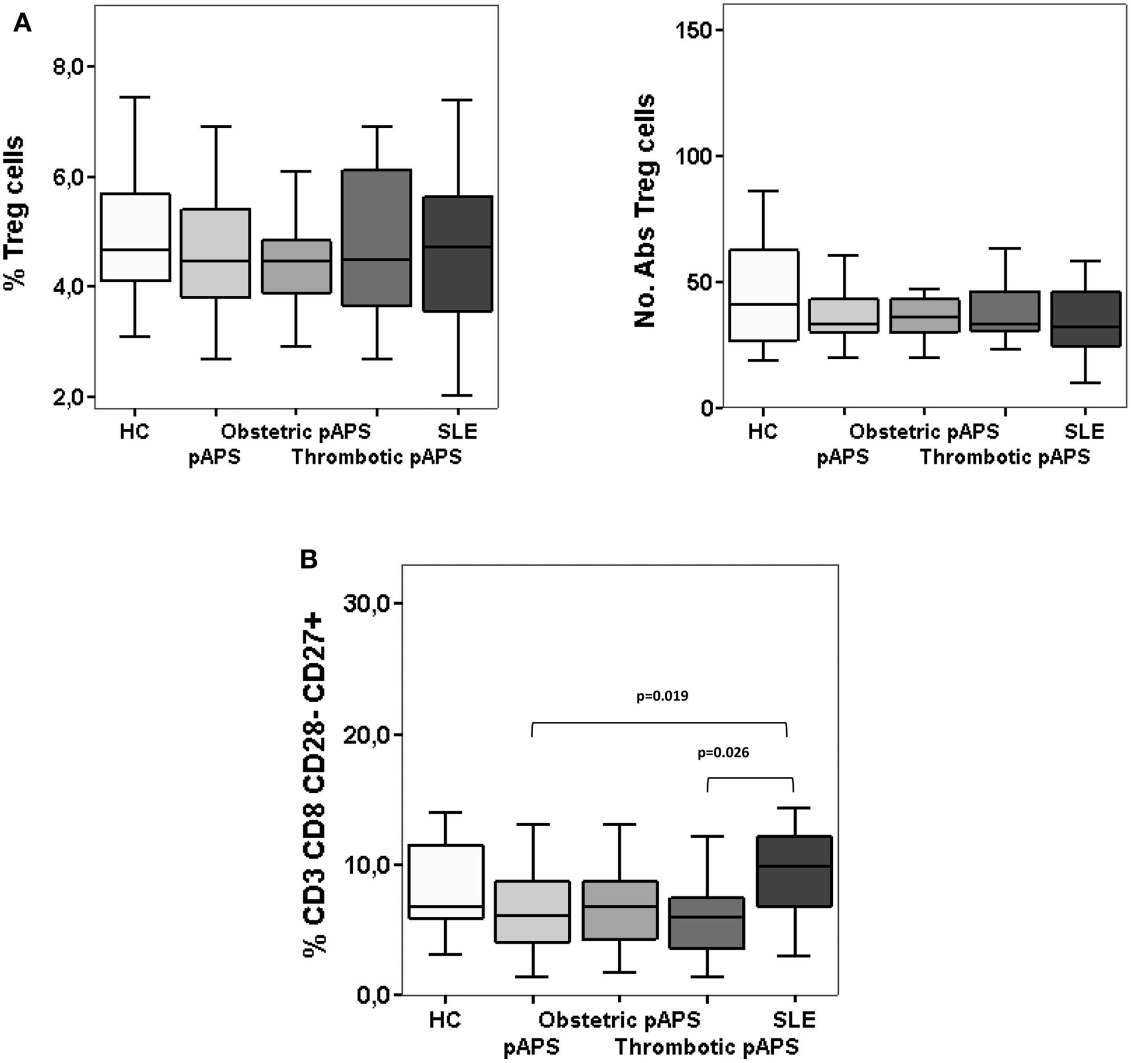
Frequencies and numbers of peripheral blood regulatory T cells: CD4^+^ CD25^hi^ CD127^−/low^ CD27^+^ CD62L^+^ CD45RO^+^ FoxP3^+^ **(A)** and CD8^+^CD28^−^CD27^+^ **(B)** cells in patients with primary antiphospholipid syndrome (pAPS, *n* = 36) and systemic lupus erythematosus (SLE, *n* = 11) and healthy controls (HC, *n* = 21). Data of pAPS patients were also analyzed according to the two pAPS variants (lower panel of figures): obstetric (*n* = 17) and thrombotic (*n* = 19) variants. Frequencies of CD8^+^CD28^−^CD27^+^ cells in pAPS patients were significantly lower than in SLE patients and mainly due to the thrombotic patients **(B)**, calculated by Mann-Whitney *U* test. There was no significant difference for CD4^+^ Tregs **(A)**. Data are expressed as the median and interquartile range. HC, healthy controls; pAPS, primary antiphospholipid syndrome; SLE, systemic lupus erythematosus.

We first looked at the main circulating regulatory T cells subtype (Tregs), identified as CD4^+^CD25^hi^CD127^low^FoxP3^+^ in fresh peripheral blood samples by flow cytometry and compared pAPS and SLE patients. No differences neither in the percentages or numbers of these circulating Tregs were found between the pAPS patients and SLE patients and neither between both disease groups and HC (Figure 1A). However, we found peripheral blood frequencies of other regulatory T cell subset, defined as CD8^+^CD28^−^CD27^+^, significantly decreased in pAPS compared to SLE patients (*p* = 0.019) but not to HC (Figure 1B). SLE patients showed no differences in the CD8^+^CD28^−^CD27^+^ compared to HC.

We subdivided pAPS patients in two groups according to their main clinical features, thrombotic or pregnancy complications. Thus, those patients with thrombotic APS had a significant decreased frequency of CD8^+^CD28^−^CD27^+^cells as compared to SLE patients (*p* = 0.026). In the case of patients with obstetric pAPS, differences did not reach statistical significance (*p* = 0.055) (Figure 1B). Frequencies and absolute numbers of peripheral blood FoxP3^+^ Tregs did not differ between the two subgroups of pAPS (Figure 1A).

### Patients With pAPS Display Decreased Frequencies of Peripheral Blood Th17 Cells Than Patients With SLE

**Figure 2:**
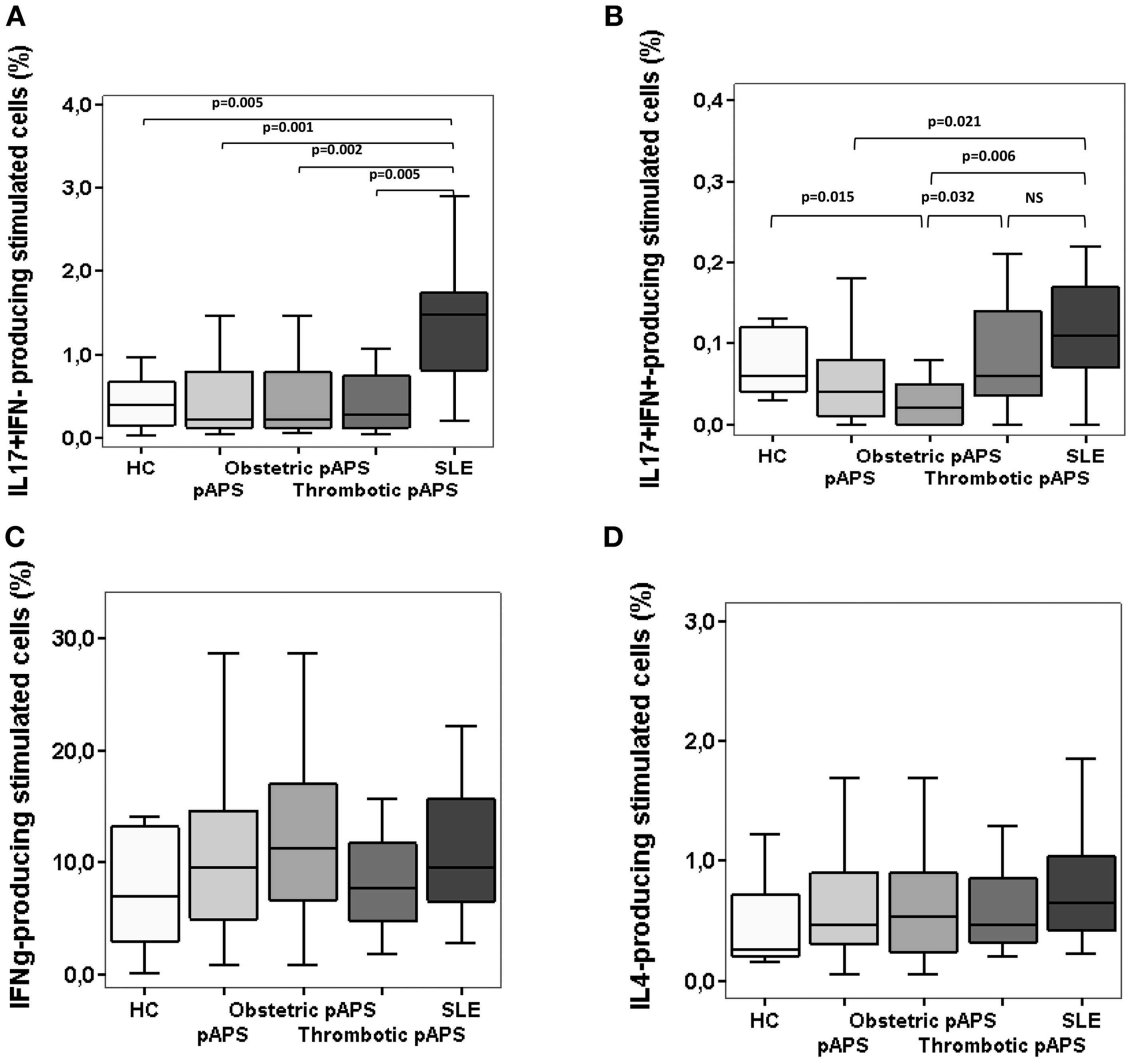
Frequencies of Th17 cells **(A,B)**, Th1 **(C)**, and Th2 **(D)** cells in patients with primary antiphospholipid syndrome (pAPS, *n* = 37) and systemic lupus erythematosus (SLE, *n* = 11) and healthy controls (HC, *n* = 21). Th17 cells were divided in two subtypes: conventional Th17 (IL-17^+^ IFN-γ^−^) and Th17Th1 (IL-17^+^ IFN-γ^+^). Data from pAPS patients were analyzed globally or separately by obstetric (*n* = 17) or thrombotic (*n* = 19) pAPS were analyzed. Only significant differences are displayed when *p* value was < 0.05 by Mann-Whitney *U*-test. Data are expressed as the median and interquartilic range and referred to the gate of CD4^+^ T cells. HC, healthy controls; pAPS, primary antiphospholipid syndrome; SLE, systemic lupus erythematosus.

Th17 and Th1 cell responses in peripheral blood from patients and healthy subjects were analyzed by measuring intracellular cytokine production by CD4+ T cells. The frequencies of Th17 cells, defined as CD4^+^CD161^+^CCR6^+^ IL17^+^IFN-γ^−^, were significantly decreased in pAPS patients, and also in HC, compared to SLE patients (*p* = 0.001 and *p* = 0.005, respectively), although no differences were found between APS vs. HC (Figure 2A). Interestingly, both patients with obstetric and thrombotic pAPS showed a lower frequency of Th17 cells compared to SLE (*p* = 0.002 and *p* = 0.005, respectively) (Figure 2B). In addition, as shown in Figure 2C, patients with pAPS had a lower frequency of CD4^+^IL-17^+^ IFN^+^ cells than SLE patients (*p* = 0.021). More specifically, those cells were also significantly decreased in patients with obstetric pAPS compared to HC, thrombotic pAPS and SLE (*p* = 0.015, *p* = 0.032, and *p* = 0.006, respectively) (Figure 2D).

Regarding Th1 response, there was a similar proportion of CD4^+^IL17^−^IFN-γ^+^ T cells in both patient groups and HC (Figure 2C). No difference was found for Th2 cells, quantified as producers of IL-4 (Figure 2D). Additionally, we also measured intracellular cytokine-producing monocytes in peripheral blood and did not observe differences in the percentages between patients and controls (Supplementary Figure 1).

### Altered Th17/Th1 and Th17/Treg Balance in SLE Patients but Not in pAPS Patients

**Figure 3:**
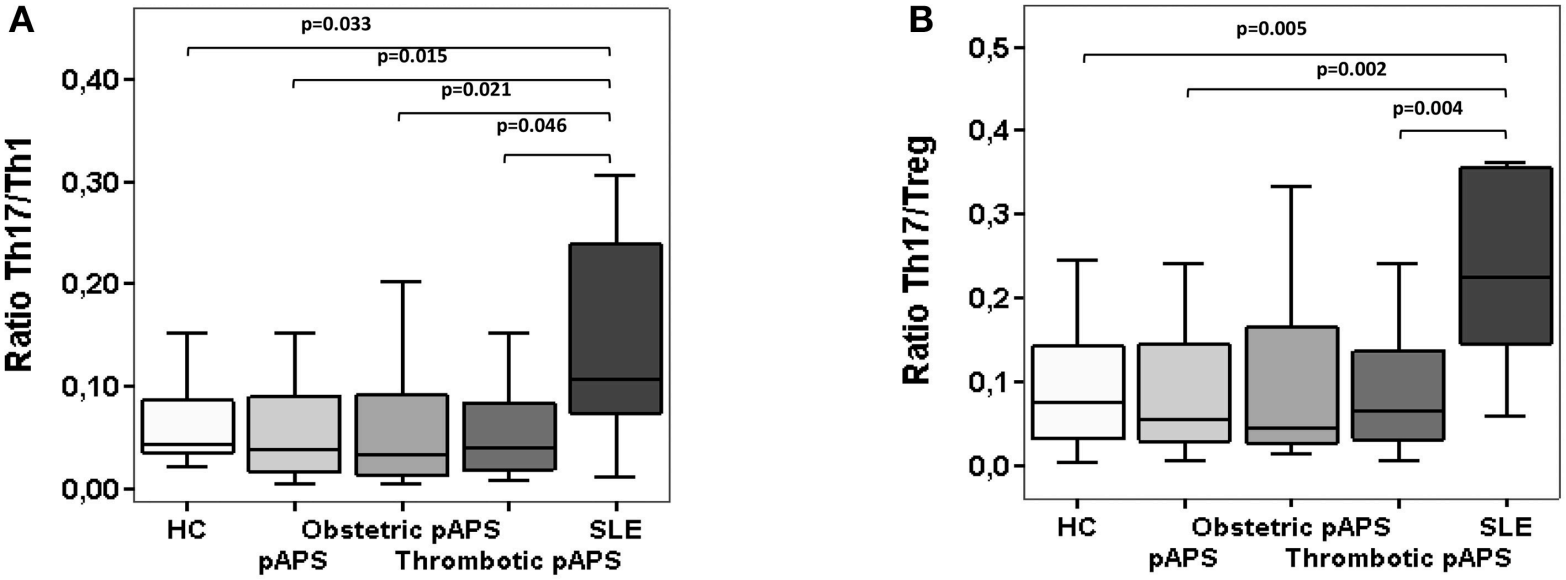
Ratios of circulating Th17/Th1 **(A)** and Th17/Treg **(B)** cells in patients with primary antiphospholipid syndrome (pAPS, *n* = 36) and systemic lupus erythematosus (SLE, *n* = 11) and healthy controls (HC, *n* = 21). Th17 were considered as T CD4+ cells IL-17^+^ IFN-γ^−^) whereas the Th1 cells were IL-17^−^ IFN-γ^+^). Treg cells were defined as CD4^+^ CD25^hi^ CD127^−/low^ CD27^+^ CD62L^+^ CD45RO^+^ FoxP3^+^. Data of pAPS patients were also analyzed according to the two pAPS variants: obstetric (*n* = 17) and thrombotic (*n* = 19) variants. The level of significant differences between groups are indicated only when *p* value was < 0.05 by Mann-Whitney *U*-test. Data are expressed as the median and interquartilic range. HC, healthy controls; pAPS, primary antiphospholipid syndrome; SLE, systemic lupus erythematosus.

The relation between Th17 cells to Th1 or regulatory T cells was determined in patients and controls by calculating the Th17/Th1 and Th17/Treg ratios. Th17/Th1 cell ratio was significantly lower in pAPS than in SLE patients (*p* = 0.015), although similar compared to HC (Figure 3A). We also noticed significant differences in the Th17/Th1 cells ratio in patients with obstetric and thrombotic pAPS compared to SLE (*p* = 0.021 and *p* = 0.046, respectively). In addition, HC had a lower Th17/Th1 cell ratio than SLE patients (*p* = 0.033) (Figure 3A).

Similarly, Th17/Treg cell ratio was significantly increased in SLE patients compared to HC and pAPS patients (*p* = 0.005 and *p* = 0.002, respectively) (Figure 3B), particularly with obstetric pAPS patients (*p* = 0.004) (Figure 3B). Again, these differences were not found in the pAPS patients compared to HC.

**Figure 4:**
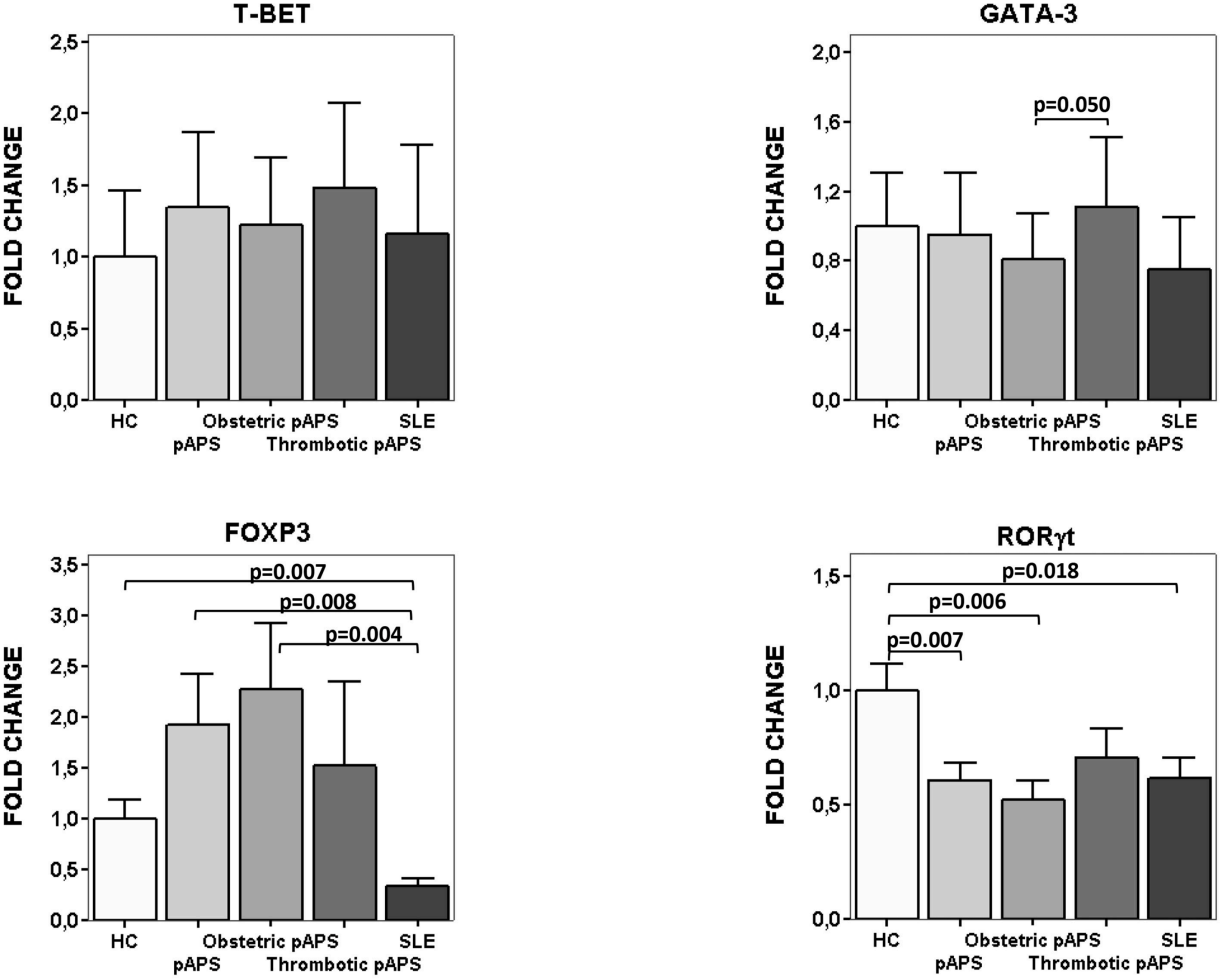
mRNA expression of transcription factors for Th differentiation. Relative fold change expression of mRNA quantified by real time quantitative PCR of T-bet, GATA-3, FoxP3, and RORγt in patients (15 pAPS patients: 8 obstetric and 7 arterial thrombosis; 10 SLE patients) and healthy controls (*n*: 10) is displayed. pAPS were subdivided into the main two variants: obstetric and thrombotic. Level of significance is only shown when *p* value was < 0.05 by Student-*t* test between groups for each gene. Data are expressed as the mean value. HC, healthy controls; pAPS, primary antiphospholipid syndrome; SLE, systemic lupus erythematosus.

We also measured the expression of specific Th transcription factors (Figure 4). SLE patients had lower FoxP3 mRNA expression levels compared to HC and pAPS (*p* = 0.007 and *p* = 0.008, respectively). In agreement with cell ratios described above, those patients with obstetric pAPS had higher FoxP3 mRNA expression levels than SLE patients (*p* = 0.004) (Figure 4). On the other hand, RORγt mRNA levels were significant reduced in SLE patients compared to HC (*p* = 0.018) but not with pAPS. Besides, pAPS patients, especially obstetric patients, had also significant reduced RORγt mRNA expression levels compared to HC (*p* = 0.006).

### Patients With APS Have Higher Serum Concentration of Immunosuppresive Molecules Than Healthy Controls

**Figure 5:**
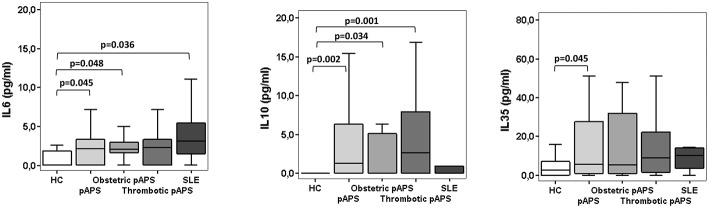
Serum cytokines levels in patients with primary antiphospholipid syndrome (pAPS, *n* = 37) and systemic lupus erythematosus (SLE, *n* = 11) and healthy controls (HC, *n* = 21). Serum IL-6 and IL-10 levels were assessed by CBA whereas serum levels of IL-35 were measured by ELISA. The level of significant differences between groups are indicated only when *p* value was < 0.05 by Mann-Whitney *U*-test. Data are expressed as the median and interquartile range. HC, healthy controls; pAPS, primary antiphospholipid syndrome; SLE, systemic lupus erythematosus.

IL-6 plays a major role in the differentiation to Th17 cells. Possibly, an excess of IL-6 could tip the balance toward the induction of Th17 cells. As shown in Figure 5, pAPS and SLE patients had higher IL-6 levels than HC (*p* = 0.048 and *p* = 0.036, respectively) but no differences were found between APS and SLE. Besides, both obstetric and thrombotic APS displayed similar IL-6 levels and not different from SLE (Figure 5). No differences in other soluble inflammatory cytokine levels such as IL-2, IL-4, TNF-α, INF-γ, and IL-17 were observed among three groups (Supplementary Figure 2).

One of the Treg-mediated suppression mechanisms is the production of immunosuppressive cytokines, such as IL-10 and IL-35. Circulating serum levels of IL10 were significantly higher in patients with pAPS than HC (*p* = 0.002). These differences were markedly increased in those patients with thrombosis (*p* = 0.001) (Figure 5). Likewise, IL-35 serum levels were also significantly increased in pAPS compared HC (*p* = 0.045) (Figure 5). Importantly, patients with SLE did not show any significant difference in these immunosuppressive molecules, although showed a trend to have lower serum levels (Figure 5).

### Normal Suppressive Capacity of Tregs in Patients With pAPS and SLE

**Figure 6:**
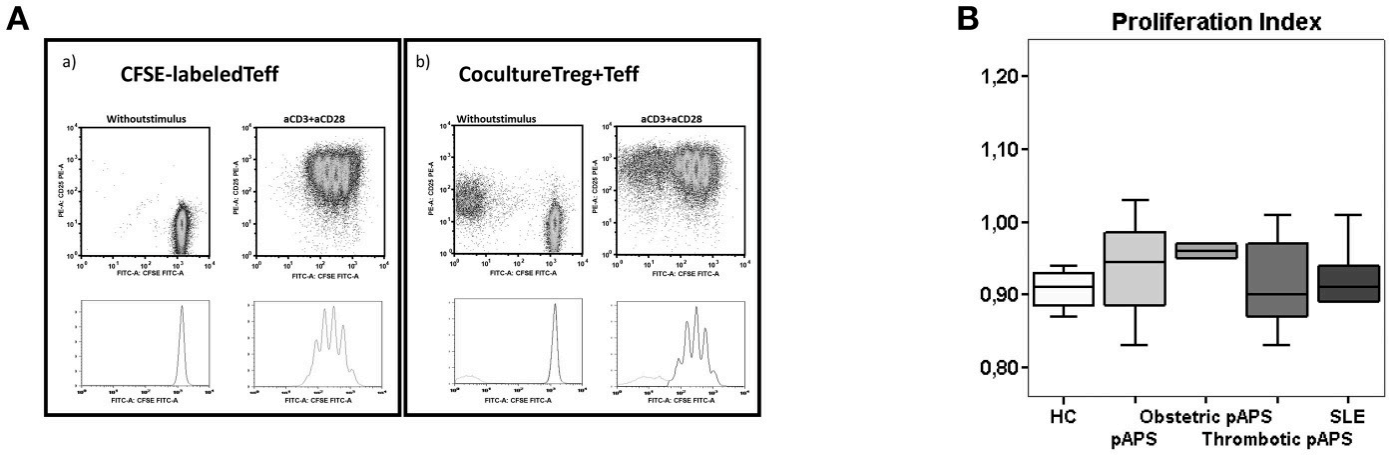
Suppression function of CD4^+^ Treg cells in *in vitro* culture with polyclonally stimulated CD4^+^ effector T cells. FACS-sorted Treg (CD4^+^ CD25^hi^ CD127^−/low^) and effector (Teff, CD4^+^CD25^−^CD127^+^) T cells of HC (*n* = 9), pAPS (*n* = 12), and SLE patients (*n* = 5) were setup in a coculture assay. Following sorting, effector T cells were labeled with 10 μM CFSE. CFSE-labeled Teff cells were cultured *in vitro* alone and cocultured with unlabeled Treg cells (1:1). Cells were either unstimulated or stimulated with anti-CD3+anti-CD28 antibodies at a bead to cell ratio 1:1 and incubated for 4 days. Then, cells were harvested and analyzed by flow cytometry. **(A)** Shows a representative experiment with only Teff cells stimulated (left) or with the addition of Treg cells to stimulated Teff cells (right). The suppressive capacity of Treg toward effector T cells in culture [ratio (1:1)/ Effect] was expressed as the proliferation index with respect to the proliferation of Teff cells stimulated without Treg cells. **(B)** Represents the median proliferation index in each group and interquartilic range.

According to ours results, we investigated the *in vitro* suppressive capacity of Tregs toward effector T cells, by co-culture assay, and the cytokine producing into culture supernatants (Figure 6).

The suppressive capacity of Tregs was similar in three study groups (Figure 6). In addition, patients with pAPS or SLE did not show differences in the concentration of suppressive IL-10 nor IL-35 (Figure 5).

## Discussion

The hypothesis of the present work is that pAPS and SLE pathogenesis may be differentiated by a distinct balance between Th17 and Tregs in peripheral blood that could result in an increased inflammatory response in SLE as compared to pAPS. There are a number of studies showing an increased inflammatory effector CD4^+^ T cells response in SLE (18–23). Treg cells have been extensively studied in peripheral blood of SLE patients, although with contradictory results (24–27). However, data of different Th subsets in pAPS are very scarce and with limited methodological approach (13, 14). Simonin et al. (28) studied the peripheral naïve, memory and effector B and T cell compartments but not Th subsets in 22 patients with pAPS and 49 controls. They found disturbances in the B cell compartment but not in T cells, which only showed a general reduction in comparison to controls. In the present work, we investigated the balance between immunoregulatory and effector immune response by measuring the number of circulating Th1, Th17, and Tregs cells as well as serum levels of cytokines in pAPS and compared them with SLE. As a limitation, the SLE patient number was low as compared with pAPS (11 vs. 37) and all of them were female what can add a bias to the analysis but it was not possible to recruit SLE male patients for the study. More importantly, we selected two well-established groups of pAPS and SLE because they had a long-term follow-up to exclude a possible progression to secondary APS from SLE, although we are aware that some authors consider pAPS and SLE as one single disease (29). Furthermore, numbers of pAPS patients were balanced between obstetric and thrombotic manifestations in the present work, in contraposition to previous studies that even do not differentiate them. Although it is possible that obstetric patients could develop thrombotic events, the time of follow-up is long-enough to differentiate both subtypes in the present work. In our experience, patients with obstetric pAPS rarely develop thrombotic pAPS (30).

Our results showed an equivalent Th17 response in pAPS to HC but decreased as compared to SLE patients. These data differ from those found by Jakiela et al. (14) that found an increased frequency of effector T CD4^+^ cells including Th17 in pAPS as compared to HC. This increase was even more evident in those pAPS patients with high titers of aPL IgG antibodies. We did not observe such an association between aPL titers and Th17 cells in peripheral blood. One possible explanation may have to be with the low serum aPL titers in our long-term patients in whom they can decrease. However, the strength of the present work is the comparison we made between pAPS and SLE patients since the latter were with low disease activity (SLEDAI ≤4) in order to exclude any influence of the systemic inflammatory response in the immunoregulatory phenotype. We also looked at the percentages of circulating IFN-γ producing Th1 cells but there was no change between both disease groups and between any disease group and HC. For intracellular cytokine-producing CD4^+^ T cells we used the percentages of cells referred to CD4^+^ T cells instead of absolute numbers, since the data were obtained after *in vitro* stimulation. Besides, there was no significant difference for absolute numbers of any lymphoid cell (CD3^+^, CD4^+^, CD8^+^, CD19^+^, and CD16^+^CD56^+^) in peripheral blood (Supplementary Figure 3) and, consequently, we considered that frequencies and absolute numbers were equivalent.

Isolated analysis of Th17 cells does not give enough information about the effector immune response since regulatory T cells may balance it (31). In this regard, peripheral blood Tregs did not differ between both disease groups and HC. This is in disagreement with other authors, that found lower frequencies of Tregs in 20 pAPS patients than in HC, although the panel of antibodies to define Tregs was shorter than the one employed in our study (32). We did not find an increase in Tregs as described by other authors in SLE (25, 26), but it could be attributed to the low disease activity of our SLE cohort. Neither, there was any significant difference in the frequency of circulating Th2 cells. As a consequence, we only observed significantly higher Th17/Tregs ratios in SLE patients than in pAPS patients and HC. Again, Th17/Tregs ratios did not differ between pAPS patients and HC. Likewise, there was a similar increased Th17/Th1 ratio in SLE. These data indicate that SLE, despite our cohort was not clinically active, showed an unbalanced inflammatory state mainly due to a higher Th17 response, with no important changes in Th1 or regulatory T cell responses. This finding was relevant since our SLE patients were mostly on antimalarial treatment, which have been described to decrease Th17-related cytokines in SLE (33). Our cellular findings were also observed for mRNA expression in PBMC. Thus, FoxP3 mRNA expression was significantly decreased in SLE as compared with HC and pAPS, as previously described (34). Such a decrease is even more significant since more SLE than pAPS patients were receiving corticosteroids what have been described as inducing factor of FOXP3 expression (34). On the other hand, ROR-gamma T expression was lower in SLE patients than in HC but not in pAPS. No important changes were detected for the other two Th master genes expression, T-bet or Gata-3. Regarding regulatory mechanisms, we did find decreased numbers of CD8^+^CD28^−^CD27^+^ cells in pAPS patients, another type of regulatory T cells different from the most studied Tregs CD4+ cells, and that has been described in mouse models and human liver transplantation (35), as well as in organ-specific autoimmune diseases (36). These cells might be induced by peripheral mechanisms and secreted suppressive cytokines, such as IL-10 or TGF-β (37). Importantly enough, there was a significant increase of the immunoregulatory IL-10 in serum from pAPS patients as compared with HC. Such a difference was not observed for SLE. To the best of our knowledge there are not report showing such a finding. The only related finding has to be not with an increase but a decrease of another regulatory cytokine, TGF-beta (14). The increase we found in serum IL-10 might support our theory of a higher immune regulation in pAPS than in SLE.

All our data together might suggest that the adaptative immune response is not significantly altered in pAPS as compared to the hallmark systemic autoimmune disease, SLE, despite pAPS is also an autoimmune response with autoantibodies production. These data differ from Xiao et al. (5) that found a diminished Th1/Th2 ratio and increased Th17 and decreased Tregs in culture of PBMC from HC with different concentrations of aPL. However, the experimental approach was different from ours since they focused on the *in vitro* effect of aPL on Th differentiation. In opposition to our findings, other authors described that β2GPI- reactive T cells might provide T cell help to B cells to induce the production of class-switched aPL in APS which was also associated with certain HLA class II genes (38). However, such a Th cell response is not specific of APS since it has been described in APS but also in SLE and in APS-negative subjects (39, 40). The specificity seems to be specific of the β2GPI epitope used to stimulate the Th cells (38). Besides, these β2GPI- reactive T cells have been found increased in APS subjects with subclinical (41) and clinical atherosclerosis (42). Interestingly, Benagiano et al. (42) evidenced domain I β2GPI- specific Th1 infiltrates in atherosclerotic plaques of pAPS patients what supported the role of the adaptative response in the induction of aPL and the plaque formation in pAPS. However, they did not studied the possible role of T follicular helper cells that provides the signal to B cells in the follicle to generate switch class and affinity maturated autoantibodies (43). In the present study, we neither focused in T follicular helper cells. Evidences suggest a higher involvement of innate response, as demonstrated by increased gene transcription of TLR8 in pAPS (11) or β2GPI-anti-β2GPI immunocomplex stimulation of monocytes through TLR4 (44). Furthermore, APS patients demonstrated increased expression of adhesion molecules in endothelium (45). On the other hand, neutrophils of pAPS patients display a pro-inflammatory gene signature (46). All the data together establish pAPS as an inflammatory autoimmune disease, although it does not induce a pro-inflammatory Th17 response as inflammation in SLE does in the present work and previous reports (18–23).

There exists a clear debate about the inflammation associated with vascular pAPS and obstetric pAPS (12). Antiphospholipid syndrome has two clinical subtypes according to obstetric or thrombotic events. From the laboratory diagnostic criteria, they do not differ and have the same aPL profile although recent evidences show also differences. In some cases, vascular and obstetric manifestations occur in the same patient, although pAPS is a stable disease in which the patient that presents with obstetric pAPS does not develop thrombosis and vice versa. This suggests that obstetric and vascular pAPS could be different diseases. Apart from clinic-epidemiological data, the study of immune-mediated mechanisms involved in both clinical variants may help to determine whether they are the same or different disease with therapeutic implication. Evidences suggest that vascular pAPS courses with complement activation that subsequently would induce thrombi, but with very low inflammation (12). On the contrary, obstetric pAPS would be induced by inflammatory mechanisms, although the presence of inflammatory cells in placenta of pAPS is very uncommon (47). Our data, although obtained from peripheral blood lymphocytes, point to a more inflammatory profile in thrombotic than in obstetric pAPS. Indeed, patients with thrombotic pAPS had a higher load of aPL than obstetric one (Table 1) and it could explain some of the differences found in Th cells between both pAPS subtypes. Furthermore, the decreased percentages of Th17Th1 cells (also considered as the subtype Th17.1) could explain the lower expression of ROR-γt, which is usually expressed in Th17Th1 cells together with T-bet (48). In any case, the number of circulating proinflammatory Th17 cells was always lower in any subtype of pAPS than in SLE. It is possible that the CD4+T cells phenotype in peripheral blood is not enough to show important differences between vascular and obstetric pAPS or, at least, does not correlate with previously described tissue cellular infiltrates in both pAPS variants.

Finally, we are aware of the limitation of our study regarding the possible effect of the different treatments in SLE and pAPS that were not considered as confounding factors. Patients with pAPS were treated with antiplatelet and/or anticoagulant therapies, whereas SLE patients were treated with antimalarials and steroids. To the best of our knowledge, we have not found any influence of treatments in pAPS on Th differentiation in the literature, although there is one study showing *in vitro* the inhibitory effect of prasugrel on effector Th cells that were induced to an inflammatory phenotype by addition of plateles (49). Patients with pAPS included in the present study did not receive prasugrel. However, the effect of antimalarials to inhibit Th17-mediated immunity is well-known (33, 50). The effect of corticosteroids in Th1 and Th17 cells has been already studied and demonstrated (51, 52). This reinforces our findings showing a stronger Th17 response in SLE than in pAPS, despite the suppressive effect of treatment.

In conclusion, our data demonstrated an increased inflammatory Th17 phenotype in patients with SLE that was not observed in pAPS patients who showed a similar phenotype to HC. In addition, thrombotic pAPS showed higher levels of peripheral blood Th17 than obstetric pAPS. Altogether, these data bring important information about the therapeutic possibilities in pAPS.

## Author Contributions

LÁ-R performed most of the experiments and participated in the writing of the manuscript. VM-T participated in research design and recruited patients. JC-A and IV participated in recruitment of patients and clinical data collection. IB contributed with analytical tools. ML-H participated in research design, supervision of experiments, and writing of the manuscript. All the authors reviewed the draft and approved it.

### Conflict of Interest Statement

The authors declare that this study received funding from Roche Farma (Spain). The funder was not involved in the study design or collection, analysis, or interpretation of the data.

## Supplementary Material

Supplementary Figure 1 Frequencies of peripheral blood monocytes secreting inflammatory cytokines in patients with primary antiphospholipid syndrome (pAPS, *n* = 37) and systemic lupus erythematosus (SLE, *n* = 11), and healthy controls (HC, *n* = 21). Data from pAPS patients were analyzed globally or separately by obstetric (*n* = 17) or thrombotic (*n* = 19) pAPS were analyzed. Monocytes from peripheral blood were intracellular stained with monoclonal antibodies specific for IL-1β, TNF-α, and IL-6, after *in vitro* stimulation with LPS for 4 hours. No significant differences were found among groups by Mann-Whitney U-test. Data are expressed as the median and interquartile range and referred to the gate of CD4^+^ T cells. HC, healthy controls; pAPS, primary antiphospholipid syndrome; SLE, systemic lupus erythematosus.

Supplementary Figure 2 Serum cytokines levels in patients with primary antiphospholipid syndrome (pAPS, *n* = 37) and systemic lupus erythematosus (SLE, *n* = 11), and healthy controls (HC, *n* = 21). Data of pAPS patients were also analyzed according to the two pAPS variants (lower panel of figures): obstetric (*n* = 17) and thrombotic (*n* = 19) variants. Serum cytokine levels (IL-2, TNF-α, IFN-γ, IL-4, IL-17) were assessed by CBA. No significant differences were found among groups. Data are expressed as the median and interquartile range. HC, healthy controls; pAPS, primary antiphospholipid syndrome; SLE, systemic lupus erythematosus.

Supplementary Figure 3 Absolute numbers of peripheral blood total lymphocytes **(A)** and lymphocyte subsets (absolute numbers in **(B)** and frequencies in **(C)**: CD3^+^, CD4^+^, CD8^+^, CD19^+^, and CD16^+^/CD56^+^) in patients with primary antiphospholipid syndrome (pAPS, *n* = 37) and systemic lupus erythematosus (SLE, *n* = 11) and healthy controls (HC, *n* = 21). Data of pAPS patients were also analyzed according to the two pAPS variants (lower panel of figures): obstetric (*n* = 17) and thrombotic (*n* = 19) variants. No significant differences were found among groups. Data are expressed as the median and interquartile range. HC, healthy controls; pAPS, primary antiphospholipid syndrome; SLE, systemic lupus erythematosus.
